# ADAPT-NMR Enhancer: complete package for reduced dimensionality in protein NMR spectroscopy

**DOI:** 10.1093/bioinformatics/bts692

**Published:** 2012-12-07

**Authors:** Woonghee Lee, Arash Bahrami, John L. Markley

**Affiliations:** ^1^National Magnetics Resonance Facility at Madison and ^2^Biochemistry Department, University of Wisconsin-Madison, Madison, WI 53706, USA

## Abstract

**Summary:** ADAPT-nuclear magnetic resonance (ADAPT-NMR) offers an automated approach to the concurrent acquisition and processing of protein NMR data with the goal of complete backbone and side chain assignments. What the approach lacks is a useful graphical interface for reviewing results and for searching for missing peaks that may have prevented assignments or led to incorrect assignments. Because most of the data ADAPT-NMR collects are 2D tilted planes used to find peaks in 3D spectra, it would be helpful to have a tool that reconstructs the 3D spectra. The software package reported here, ADAPT-NMR Enhancer, supports the visualization of both 2D tilted planes and reconstructed 3D peaks on each tilted plane. ADAPT-NMR Enhancer can be used interactively with ADAPT-NMR to automatically assign selected peaks, or it can be used to produce PINE-SPARKY-like graphical dialogs that support atom-by-atom and peak-by-peak assignment strategies. Results can be exported in various formats, including XEASY proton file (.prot), PINE pre-assignment file (.str), PINE probabilistic output file, SPARKY peak list file (.list) and TALOS+ input file (.tab). As an example, we show how ADAPT-NMR Enhancer was used to extend the automated data collection and assignment results for the protein *Aedes aegypti* sterol carrier protein 2.

**Availability:** The program, in the form of binary code along with tutorials and reference manuals, is available at http://pine.nmrfam.wisc.edu/adapt-nmr-enhancer.

**Contact:** whlee@nmrfam.wisc.edu or markley@nmrfam.wisc.edu

## 1 INTRODUCTION

One of the goals of protein nuclear magnetic resonance (NMR) spectroscopy is to increase its throughput by automating the steps of data collection, spectral assignment and structure determination. The latest approach towards this goal from our laboratory is ADAPT-NMR (Bahrami *et al.*, 2012), a software package that interfaces with the NMR spectrometer and uses an algorithm for devising a pathway for optimal data collection to approach the goal of complete data assignment. As new data are collected, ADAPT-NMR analyzes the set of data collected up to that point and chooses the next step for data collection. Each data collection step involves choosing a 3D NMR experiment and a particular tilted plane that will identify peaks in the 3D spectrum. ADAPT-NMR incorporates an earlier approach to fast data collection, HiFi-NMR (Eghbalnia *et al.*, 2005) and an algorithm for automated probabilistic assignment, PINE-NMR (Bahrami *et al.*, 2009). The output from ADAPT-NMR is a probabilistic assignment table and analysis of secondary structure. As a means for visualizing the spectral data, picked peaks and spin system assemblies underlying these assignments, we have developed the standalone software package described here, ADAPT-NMR Enhancer.

## 2 IMPLEMENTATION

**Fig. 1.**
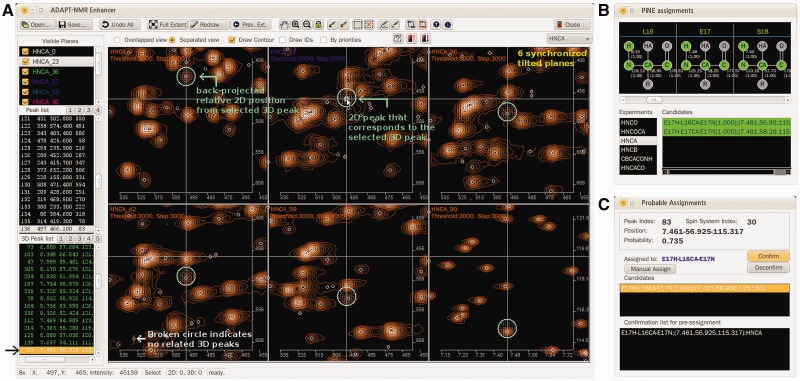
ADAPT-NMR Enhancer user interface with AeSCP-2. (**A**) *Main Window Dialog* for tilt plane visualization. (**B**) *PINE Assignment Dialog* for atom-by-atom assignment. (**C**) *Probable Assignment Dialog* for peak-by-peak assignment

ADAPT-NMR Enhancer is an SDI (Single Document Interface) application written in C++ with QT4 libraries (http://qt.nokia.com) for graphical user interface. The software supports multiple operating systems (MS Windows, MacOSX and Linux). ADAPT-NMR Enhancer offers three active dialog boxes: *Main Window Dialog*, *PINE Assignment Dialog* and *Probable Assignment Dialog*. The *Main Window Dialog* (Fig. 1A) allows the visualization of peaks picked in 2D tilted planes and their positions in 3D space. 2D and 3D peak lists are located to the left of the dialog box; file I/O (input/output), visual manipulation, peak picking, linking and assignment tools are located at the top of the dialog box. A maximum of six synchronized 2D tilted planes can be viewed at once. The *x*-axis represents the ^1^H chemical shift dimension, which is invariant with tilt angle. However, the *y*-axis is a combination of ^13^C and ^15^N chemical shifts as represented by the tilt angle. Thus, it is hard for users to judge the correctness of 3D peaks constructed from peaks in tilted 2D planes. ADAPT-NMR Enhancer offers two functions to resolve this problem. When one chooses a constructed 3D peak from the 3D peak list at the left side of the dialog box, circles appear in the displayed 2D tilted planes at positions where peaks are expected, and a lime-colored dot identifies peaks associated with the 3D reconstruction (Fig. 1A). Alternatively (not shown), the user can right-click and drag a 2D peak to give it the lime dot and identify the corresponding peak in the 3D peak list; again, regions in the displayed 2D planes where peaks are expected are circled. Tools located at the top of the *Main Window Dialog* can be used not only to validate the automated peak picking and assignment but also to add missing peaks, remove peaks picked in error or correct assignments. PINE-SPARKY (Lee *et al.*, 2009) tools have been incorporated into ADAPT-NMR Enhancer to assist with resonance assignments. The *PINE Assignment Dialog* (Fig. 1B) displays the peptide chain with atoms associated with assigned chemical shifts with their probabilities indicated by color coding. The candidate list box shows all 3D resonances for a given experiment that PINE considered as possible assignments for the selected atom. If the constructed 3D spectrum does not exhibit the predicted peak, the user can examine the linked 2D tilted planes for evidence of a peak. This examination is accomplished by double-clicking the candidates, so as to view the corresponding 3D peak. The Probable Assignment Dialog box pops up when a 3D peak from the 3D list box or from the spectral view is selected. It lists possible assignments for a peak along with their probabilities. The PINE Assignment Dialog box is based on atoms, whereas the Probable Assignment Dialog box is based on peaks. The user can either confirm or modify the assignment for a 3D peak. The decision is stored in the confirmation list box, and the results can be exported in a variety of file formats.

## 3 RESULTS AND CONCLUSION

AeSCP-2 (110 residues) is the *Aedes aegypti* sterol carrier protein 2, which is involved in cellular lipid transport mechanisms related to lipid uptake and metabolism (Gallegos *et al.*, 2001; Singarapu *et al.*, 2010). This protein was the one used to test ADAPT-NMR (Bahrami *et al.*, 2012). Although assignments were made to 510 atoms with >99% probability of correctness, the assignment probabilities of 24 atoms was ≤99% and no assignment were obtained for 5 atoms. We used ADAPT-NMR Enhancer to visualize and improve the quality of the assignments. We manually added peaks that had not been picked by the automated algorithm; we deleted picked peaks clearly arising from noise; and we modified the priority scores of the peaks on the basis of manual assessment. With the new peak set as input, ADAPT-NMR yielded improved scoring: of the 24 assignments initially scored at <99% probability, only 7 remained <99% probability. We then used the manual features of ADAPT-NMR-Enhancer to determine why these seven assignments were of lower probability. We found, for example, that because residue 80 is proline, the CBCA(CO)NH dataset yielded no connectivities from P80 to the CA and CB of L79. However, we could easily confirm the assignment from HNCA(HNCB) data. Another atom with low-assignment probability, A60CA, was found to have a low-peak intensity that prevented its detection in the CBCA(CO)NH experiment. The missing peak was easily added by using the editing tool of ADAPT-NMR Enhancer, so that ADAPT-NMR recognizes the peak when the program is re-run. All backbone resonance assignments were confirmed or completed by means of a ‘sequential walk’ through the 3D HNCA(HNCB) and CBCA(CO)NH data. The ‘Lock’ tool in ADAPT-NMR Enhancer, which enables one to predict the position of a 3D peak by selecting two peaks from 2D tilted planes, was found to be useful in confirming assignments. In cases where a large number of noise peaks have been deleted, ADAPT-NMR will suggest another experiment and tilt angle for data collection. The detailed strategies used are documented at http://pine.nmrfam.wisc.edu/adapt-nmr-enhancer.

*Funding*: National Center for Research Resources (5P41RR002301-27) and National Institute of General Medical Sciences (8 P41 GM103399-27) from the National Institutes of Health.

*Conflict of Interest*: none declared.
